# Environmental Controls to Soil Heavy Metal Pollution Vary at Multiple Scales in a Highly Urbanizing Region in Southern China

**DOI:** 10.3390/s22124496

**Published:** 2022-06-14

**Authors:** Cheng Li, Xinyu Jiang, Heng Jiang, Qinge Sha, Xiangdong Li, Guanglin Jia, Jiong Cheng, Junyu Zheng

**Affiliations:** 1 National-Regional Joint Engineering Research Center for Soil Pollution Control and Remediation in South China, Guangdong Key Laboratory of Integrated Agro-Environmental Pollution Control and Management, Institute of Eco-Environmental and Soil Sciences, Guangdong Academy of Sciences, Guangzhou 510650, China; xyjiang@soil.gd.cn (X.J.); hjiang@soil.gd.cn (H.J.); xdli@soil.gd.cn (X.L.); chengjiong@soil.gd.cn (J.C.); 2 Institute for Environmental and Climate Research, Jinan University, Guangzhou 511443, China; shaqinge2021@jnu.edu.cn (Q.S.); jiaguanglin@jnu.edu.cn (G.J.); zheng.junyu@gmail.com (J.Z.)

**Keywords:** heavy metal, soil pollution, factorial kriging, multiple scales

## Abstract

Natural and anthropogenic activities affect soil heavy metal pollution at different spatial scales. Quantifying the spatial variability of soil pollution and its driving forces at different scales is essential for pollution mitigation opportunities. This study applied a multivariate factorial kriging technique to investigate the spatial variability of soil heavy metal pollution and its relationship with environmental factors at multiple scales in a highly urbanized area of Guangzhou, South China. We collected 318 topsoil samples and used five types of environmental factors for the attribution analysis. By factorial kriging, we decomposed the total variance of soil pollution into a nugget effect, a short-range (3 km) variance and a long-range (12 km) variance. The distribution of patches with a high soil pollution level was scattered in the eastern and northwestern parts of the study domain at a short-range scale, while they were more clustered at a long-range scale. The correlations between the soil pollution and environmental factors were either enhanced or counteracted across the three distinct scales. The predictors of soil heavy metal pollution changed from the soil physiochemical properties to anthropogenic dominated factors with the studied scale increase. Our study results suggest that the soil physiochemical properties were a good proxy to soil pollution across the scales. Improving the soil physiochemical properties such as increasing the soil organic matter is essentially effective across scales while restoring vegetation around pollutant sources as a nature-based solution at a large scale would be beneficial for alleviating local soil pollution.

## 1. Introduction

Soil heavy metal pollution has been threatening food security and human health all over the world with the increasing intensification of urbanization, industrialization, and population growth [1]. During the past decades, both urban and agricultural soils have suffered from heavy metal pollution in China, especially for cities with rapid urbanization [2,3,4,5]. As reported, the soil heavy metal pollution in 32 Chinese cities was greatly elevated (see the review by [4]). For pollution mitigation and health protection opportunities, it is critical to identify the spatial variations and environmental drivers of soil heavy metal pollution in cities [6,7,8].

Soil heavy metal pollution is distributed unevenly across spatial scales. The soil pollution in space is an outcome of several natural and anthropogenic processes performing at multiple spatial scales [9]. Different influencing factors generally have different spatial spreads [10]. Some of the influence is more likely to be observed at site- or short-distances (e.g., sewage irrigation, agrochemical additions, and traffic or mining or industrial emissions, etc.) [11,12,13] whereas others such as the weathering of parent materials is prone to be observed at larger distances [9]. The combined effect of different influencing factors operating at different spatial scales yielded nested/mixed information for soil pollution from different scales [14]. Identifying the spatial variability of soil pollution at distinct scales and scale-related influencing factors is critical for mitigation purposes [10].

Traditional multivariate statistical analyses (i.e., correlation, partial redundancy analysis) were commonly used to explore the critical factors influencing soil pollution. Previous studies used these methods and found that many environmental variables such as soil properties, landscape pattern and changes, parent material composition, and distance to potential pollution sources were shown to be closely correlated with soil heavy metal pollution [1,9,15]. The key environmental drivers of soil pollution depended on spatial scale and heavy metals [16]. However, traditional statistics might blur the correlations from several different spatial scales and thus are unable to discern the combined effect of different interrelationships [17]. Furthermore, traditional statistics are likely to cause bias in the real correlations due to the spatial auto-correlations inherent in space [15]. Due to Tobler’s first geographic law, things close to each other are more closely correlated, and spatial autocorrelation is ubiquitous.

Factorial kriging analysis (FKA), a geostatistical method, which was initially proposed by Matheron [18], and subsequently developed by Wackernagel [19] and Goovaerts [14,20], allows for the investigation of the spatial autocorrelation and discriminating spatially structured variance of the variables. FKA assumes that structurally nested information could be decomposed into several independent spatial components, indicating the underlying processes acting on different scales using variogram and cross-variogram analyses. Spatial components are data processing artifacts without any physical meaning. However, they help identify the key drivers in determining the spatial variation of variables based on the scale they operate and enhance the relations among variables blurred or mixed by a traditional statistical analysis. FKA has been used in soil sciences including soil properties and soil pollution [17,21,22,23]. For example, using factorial kriging and stepwise regression, a previous study found that the driving forces for variations in soil Cd, Pb, and Zn contents changed from land use types at a short-range scale to pedogenesis at a long-range scale [23].

Many previous studies have noticed the spatial autocorrelation and variability of the soil pollution variables [23,24]. However, few studies have considered the spatial autocorrelation and structure variance of the environmental factors, let alone explored the complex interactions between the soil pollution and environmental factors on various spatial scales. A geostatistical and multi-scale approach is needed to quantify the soil pollution process in highly urbanized regions and provides information for effective pollution mitigation and management.

Therefore, the main purpose of this study is to discriminate the variance of soil heavy metal pollution across multiple spatial ranges and the underlying processes acting on each scale in a rapidly urbanized city of Guangzhou, South China. Guangzhou has experienced elevated soil heavy metal pollution during the past few decades, which is an ideal place to study. We proposed two research questions: (1) how does the variance of soil heavy metal pollution change across different spatial ranges (scales)? and (2) which of the environmental variables (i.e., climate, soil properties, socioeconomic factors, distance to potential sources, atmospheric heavy metal emissions) dominantly influenced the spatial variance of soil pollution at different scales (spatial ranges)? We hypothesized that soil pollution with heavy metals was mainly determined by the soil physicochemical properties at short distances as they influenced the patchy inputs of heavy metals. Atmospheric deposition and lithological processes as indicated by atmospheric heavy metal emissions and the distance to pollutant sources, climate, and socioeconomic factors dominated the soil pollution at long distances.

## 2. Materials and Methods

### 2.1. Study Area

**Figure 1:**
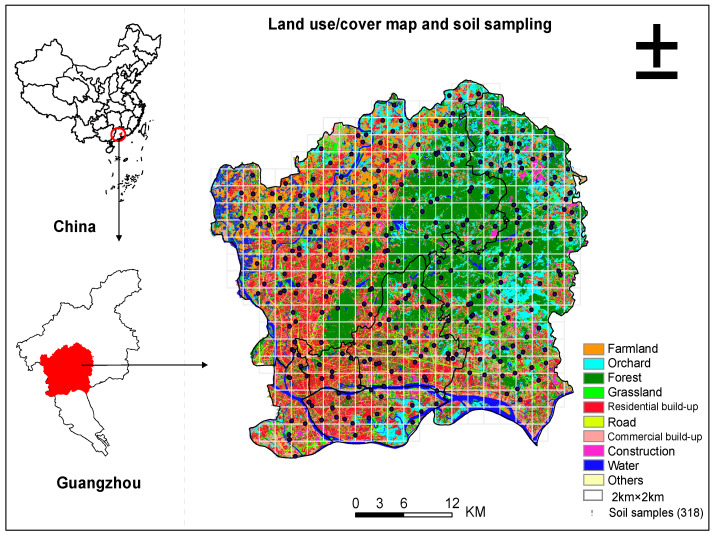
The location, land use, and cover map and soil samples.

Our study area was in central Guangzhou City, the capital of Guangdong Province in South China (112°57′–114°3′ E, 22°26′–23°56′ N) (Figure 1). Guangzhou covers about 7434 km^2^ and has experienced rapid urbanization and industrialization since the 1980s with a population of 14 million and a GDP of 294 billion US dollars in 2016 [25]. It has a subtropical monsoon climate with an annual temperature and precipitation of 20–22 °C and 1700 mm, respectively. The topography of the study area is high in the northeast while low in the southwest, with the main landform types of hilly lands in the north and east and alluvial plains in the south. There are six soil groups in the study area including paddy soils (accounting for 50%), lateritic red earths (40%), vegetable soils (8%) as well as fluvo-aquic soils, litho soils, and artificial soils (2% in total for the latter three). Alluvial deposits and granite are the primary parent materials in the study area [1].

### 2.2. Soil Sampling and Chemical Analysis

We applied a random tessellation design (one random point in each 2 km × 2 km grid) to collect soil samples during 14–16 October 2015 and 2–30 November 2016. In total, 318 topsoil (0–20 cm) samples were collected in different land use and land cover types with each soil sample mixed with 5–8 subsamples (Figure 1). All samples were air dried, stones and debris were removed, and subsequently sieved with nylon sieves (10 and 100 mesh) for further soil property and heavy metal extraction analyses. Soil pH was measured by potentiometry (soil:water, 1:2.5 by mass). Soil texture and organic matter were measured by the hydrometer method and potassium dichromate oxidation volumetric method, respectively. For heavy metal extraction, the inductively coupled plasma atomic emission spectrometry (ICP-AES) was used for Cd, Cr, Cu, Ni, and Pb, while hydride atomic fluorescence spectrometry for As, and atomic fluorescence spectrophotometry (AFS) for Hg. The Chinese standardized reference material (GSS24) replicates and blank correction were used for the accuracy control. The measurement accuracy was less than 4%, while the recovery rate of heavy metals was between 96% and 102%. More detailed information about the soil sampling scheme and the extraction of the soil physiochemical properties are provided in [1].

### 2.3. Attribute Analysis

#### 2.3.1. Environmental Factor Selection

According to previous studies on soil heavy metal pollution [16] and data availability, we used the geo-accumulation index (i.e., I_geo_) to represent soil heavy metal pollution [9] and selected five types of environmental drivers of soil heavy metal pollution. That is: (1) the soil properties in terms of pH, organic matter (SOM), and texture composition (i.e., clay, sand and silt) from the soil samples; (2) the biophysical factor of NDVI (1 km × 1 km; http://www.resdc.cn, accessed on 9 October 2018); (3) the pollution source influence as indicated by the distance to the potential pollutant source (e.g., road, river and industry) in 2013 (DisRd, DisRv, DisInd; [1]); (4) the socioeconomic factors of GDP and Population (Pop) from IGR-CAS (1 km × 1 km; http://www.resdc.cn, accessed on 9 October 2018); (5) the climate factors of the annual mean precipitation (Prec) and temperature (Temp) from IGR-CAS (1 km × 1 km; http://www.resdc.cn, accessed on 9 October 2018); (6) the atmospheric heavy metal emissions estimated by the emission inventory [26] (1 km × 1 km). For the variables that generally had negative correlations with soil pollution, we used their reciprocal forms (calculated by 1/(x + 0.01)) (i.e., sand*, NDVI*, DisRd*, DisRv*, DisInd*) to later explicitly show their close relationships with soil pollution.

#### 2.3.2. Data Pre-Processing (Gaussian Anamorphosis)

To avoid the bias from the multi-scale data and observation outliers, the Gaussian anamorphosis transformation procedures were employed to transform all of the observations to Gaussian distributions [23]. Before the Gaussian transform, the outliers for the soil heavy metal concentrations were removed using the three times standard deviation criteria (mean ± 3SD). Finally, there were 280 quality soil samples for further analysis.

#### 2.3.3. Multivariate Factorial Kriging Analysis

Spatial autocorrelation could be fitted by the semi-variogram model with the semi-variance as the *Y*-axis while the sampling interval *h* was the *X*-axis. The distance where the model first flattened is known as the range, and the samples are no longer correlated beyond the range, while the semi-variance value corresponded to the *Y*-axis at the distance of the range is called the sill. The semi-variance value at zero separation distance (i.e., *h* = 0) is called the nugget. Theoretically, the nugget is 0. However, the nugget is larger than 0. The discontinuity of the semi-variogram at a zero separation distance, known as the nugget effect, is due to the spatial variation caused by a small sampling interval or measurement error, or both [14].

The multivariate factorial kriging analysis (MFK) has been widely used to study the spatial variance and sources of soil heavy metals across multiple scales [21,22,23,27]. It combined the variogram model and principial component analysis to identify the variance and sources of soil heavy metals at distinct spatial scales.

The MFK identifies the scale of variability by the variograms and cross variogram fitting curves calculated based on the experimental data.

(1) $$\gamma_{uv}\left(h\right)=\frac{1}{m}\overset{m}{\underset{j=1}{\sum }}\left[Z_{u}\left(x_{j}\right)-Z_{u}\left(x_{j}+h\right)\right]\left[Z_{v}\left(x_{j}\right)-Z_{v}\left(x_{j}+h\right)\right]$$

where $\gamma_{uv}\left(h\right)$ is the semivariance for variables $Z_{u}\left(x\right)$ and $Z_{v}\left(x\right)$ for the sampling distance interval (i.e., lag size) of *h*; m represents the number of pairs of the two variables; *Z*$\left(x_{j}\right)$ and *Z*$\left(x_{j}+h\right)$ represent the measurements at the distances *x* and *x* + *h*, respectively. The function (1) calculates the variogram (*u* = *v*) or cross variograms (*u* ≠ *v*).

In this study, eight heavy metals and eighteen environmental factors yielded a total of 351 variograms and cross-variograms. The omnidirectional variograms were calculated due to the small anisotropy ratio (<2.5 [17]). We used the variograms and cross-variograms to identify the scale of variability for variables based on turning points of the curves.

Using a linear model of coregionalization (LMC), the total variance of the variables could be functioned as a linear combination of proportional covariance (i.e., variogram) at multiple scales:

(2) $$\gamma_{ij}\left(h\right)=\overset{n}{\underset{j=1}{\sum }}B_{ij}{}^{n}g_{ij}{}^{n}$$

where $\;\gamma_{ij}\left(h\right)\;$ is the variance for the *i*th variable on the *j*th spatial scale at the lag size of *h*; $b_{ij}{}^{n}$ is a coregionalization matrix characterizing the interrelationships among the variables at the *n*th scale; $g_{ij}{}^{n}$ is the basic variogram for the *n*th scale. Spatial structures characterized by the spatial components of heavy metals at different scales could be obtained by the ordinary cokriging method. The optimal combination of the variogram fitting models at multiple scales could be determined by two statistical parameters of the residual sum of square and the Akaike information criterion. The smaller the values of the two indicators, the more appropriate the LMC fitting model. Cross validation was conducted by the leave-one method. That is, one point was subtracted in sequence and the remaining points were used to estimate the value at a certain location. Two indicators (i.e., mean error, ME; root mean squared standardized errors, MSSE) were used to assess the accuracy of the LMC fitting. When the ME was closer to 0 and the MSSE was closer to 1, the better the model fit.

Principal component analysis (PCA) was performed at each scale to explore the environmental determinants for soil pollution. The correlation coefficients $\rho_{\mathrm{ij}}$ of the variables on the *j*th scale was calculated as follows [23]:

(3) $$\rho_{\mathrm{ij}}=q_{\mathrm{ij}}\sqrt{\lambda_{j}/\sigma_{i}}$$

where $q_{\mathrm{ij}}$, $\lambda_{j}$, and $\sigma_{i}\;$ represent the corresponding value in eigenvector, the eigenvalue, and variance of the *i*th variable, respectively.

**Figure 2:**
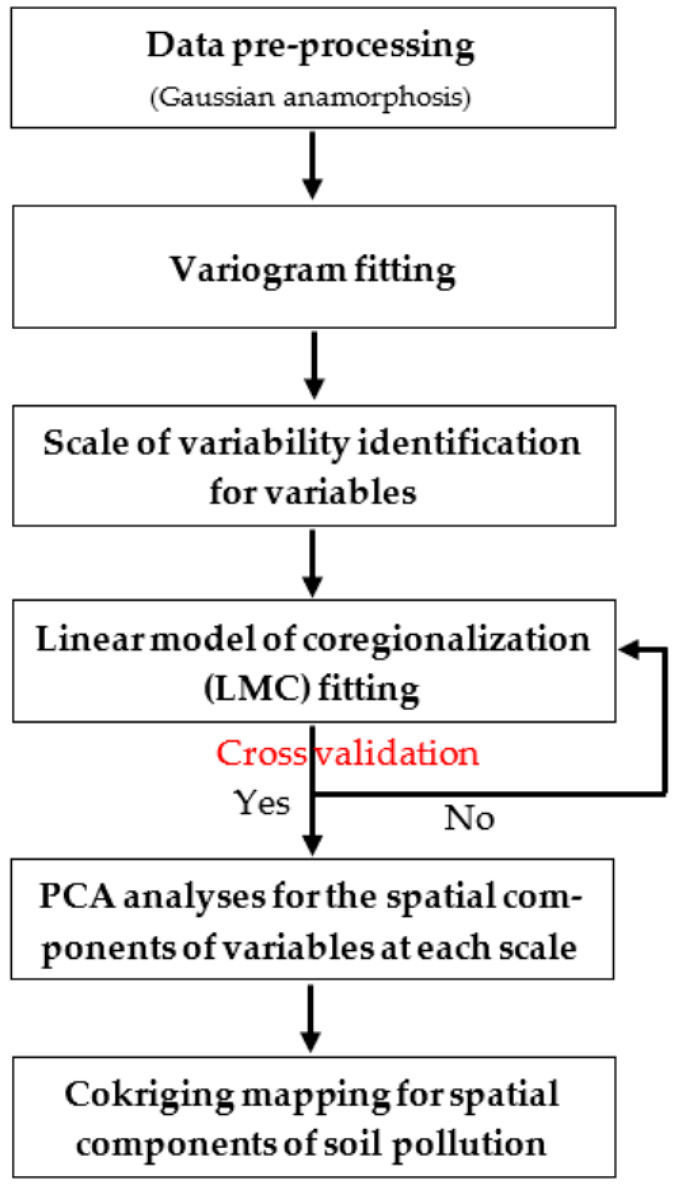
A flowchart of the multivariate factorial kriging analysis.

The flowchart of MFA is shown in Figure 2. All of the geostatistical analyses (LMC fitting, co-kriging, cross-validation) were performed using ISATIS software (Geovariances and the Center of Geostatistics of the Paris School of Mines, Fontainebleau, France; version: 2016).

## 3. Results

### 3.1. Soil Pollution Characteristics and Its Relations with Environmental Factors

The concentrations of heavy metals were strongly right skewed with many large values and few extremely low ones (skewness > 0). The mean and standard deviation of heavy metal concentrations (mg/kg) was 10.07 ± 9.94, 0.32 ± 0.64, 33.75 ± 28.10, 28.22 ± 36.40, 0.27 ± 0.47, 15.80 ± 18.78, 54.29 ± 39.93, and 110.25 ± 80.29 for As, Cd, Cr, Cu, Hg, Ni, Pb, and Zn, respectively. The coefficients of variance for the contents of heavy metals were between 0.73 and 1.99, showing a high variability. Compared to the Guangdong background values [28], 18–88% of the soil samples were polluted to some extent, as indicated by the geo-accumulative indices of soil heavy metal pollution (Table S1). The mean and median of the heavy metal concentrations for Cd, Cu, Hg, Ni, Pb, and Zn were 0.4–3.5 times higher. However, compared to the screening values for the agricultural soils (GB15618-2018) and the soils of development land (GB36600-2018), 12.6% of the samples for Cd exceeded the standard, indicating their potential hazard to the ecosystem and human health, while it was 6.6% for Pb, 4.7% for As, 4.4% for Cu, 4.1% for Zn, and below 2% for Hg, Cr, and Ni.

To gain a general understanding of the soil pollution and environmental factors, we conducted a Pearson correlation analysis based on the Gaussian-transformed variables. The correlation coefficients indicated that most of the variables were significantly correlated (Table S2). The soil heavy metal pollution was positively correlated with most of the soil physicochemical properties, socioeconomic factors, temperature, and atmospheric heavy metal emissions at the *p* < 0.05 level. Negative correlations were found between soil pollution and the distance variables (i.e., DisInd, DisRd) as well as the NDVI, as indicated by the positive correlations with their reciprocals. We noted that the correlation coefficients for most pairs of variables were relatively low (<0.6), indicating weak correlations between the soil pollution and environmental factors.

### 3.2. LMC Fitting Results

We focused on the variogram fittings for the eight heavy metals. The variances for the heavy metals increased sharply as the lag distance increased up to 3 km, and then steadily increased up to 12 km (Figure S1). A few variances for the cross-variograms of the heavy metals decreased with the increasing lag distance (e.g., Cr–Hg, Cu–Hg, Hg–Pb, Hg–Ni, Hg–Zn), indicating different spatial variations in these variable pairs. Generally, the total variance of soil heavy metal pollution could be functioned as a linear combination of a nugget effect, and a short-range (3 km) variance and a long-range (12 km) variance by two spherical variogram models due to the least sum of squared residuals and the Akaike criterion for the LMC fitting. The cross validation showed that the absolute values of the MEs and MSSEs for soil pollution with heavy metals close to 0 (−0.020–0.015) and 1 (0.93–1.11; Pb: 1.21), implying a good accuracy of LMC fitting for the soil contaminants.

### 3.3. Interrelationships and Drivers of Soil Heavy Metals at Multiple Scales

The soil heavy metal pollution showed either enhanced or counteracted correlations with environmental factors across the spatial scales (Table S3). The soil physicochemical properties such as sand*, silt, and clay generally showed consistent positive correlations with soil pollution with heavy metals. Other variables showed scale-dependent correlations with soil pollution, for example, the correlations between soil pollution with the most heavy metals and the Temp, NDVI*, socioeconomic factors of GDP and Pop, the distance to pollutant sources of DisRd*, DisRv*, DisInd*, and the atmospheric emissions variables changed from negative at a short structure to positive at a long structure. Correlations between most heavy metal pollution and SOM changed from positive at the nugget and short-range scales to negative at the long-range scale. These kinds of counteractions of the correlations on various scales resulted in relatively weak correlations among the variables by the traditional correlation analyses shown in Table S2.

**Figure 3:**
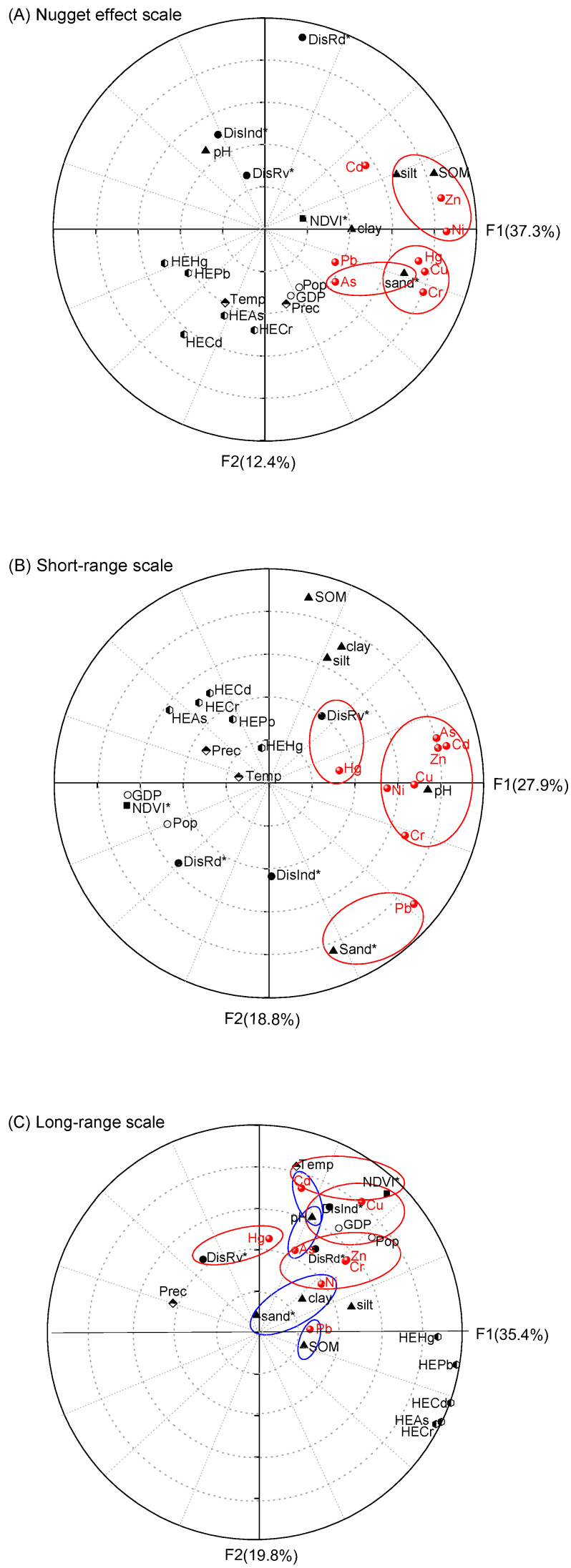
Projections of the correlations between the spatial components for the heavy metals and the principal component scores into unit circles at the nugget (**A**), short-range (**B**), and long-range (**C**) scales. The variables within circles in red or blue indicate close correlations amongst each other.

To clearly show the scale-dependent interactions among variables, principal component analyses were conducted on the regionalized variables at each scale. We projected the correlations between the first two principal components (i.e., PC) and all the variables into a unit circle at each scale for better visualization (Figure 3). At the nugget effect structure, 49.7% of the total variance of soil heavy pollution could be explained by PC1 (37.3%) and PC2 (12.4%). PC1 showed a high and positive correlation with sand*, SOM, silt, Cr, Cu, Hg, Ni, and Zn, indicating the dominant influence of these variables at this PC. PC2 showed strong and positive correlations with DisRd*, indicating the influence of traffic activities.

At the nugget scale, three groups were found, as indicated by the unit circle and the correlation coefficients (Table S3). The first group included soil pollution with Ni and Zn and SOM and silt, while the pollution with Cr, Cu, and Hg and sand* formed the second group, and the As and sand* formed the third group. However, the relatively strong associations between Cd and Temp, and Pb and Prec, DisInd*, and HEcr, as indicated in Table S3, were not inflected by the unit circle (Figure 3). Atmospheric emissions for heavy metals were generally correlated with climatic factors of Temp (Figure 3). Obviously, soil pollution with the most heavy metals were notably influenced by the soil physicochemical properties.

At the short-range scale (3 km), the PC1 and PC2 in total explained 46.7% of the structure variance (PC1: 27.9%, PC2: 18.9%). PC1 had a strong positive correlation with pH and As, Cd, Cr, Cu, Ni, and Zn pollution in the soil, indicating the dominant influence of pH on soil contamination. PC2 had a strong and positive correlation with SOM, clay, and silt, and a negative correlation with sand* and Pb, indicating the determination of these soil properties on Pb. Similarly, soil pollution with Hg was highly correlated with DisRv*, indicating the potential influence of sewage irrigation. Compared to the general correlation at this spatial structure (Table S3), the strongly negative correlations between several pairs of correlations were not detected in the unit circle: (1) As and Cd and GDP and Pop; (2) As, Cr and Hg and NDVI*; (3) Cr and HECr. This indicated that the soil physiochemical properties determined the soil pollution with nearly all of the heavy metals, while the socioeconomic factors, vegetation cover, and atmospheric emissions also played an important role for a few metals at a short-range structure.

At the long-range structure (12 km), the first two PCs explained 55.2% of the structure variance (PC1:35.4%, PC2: 19.8%). PC1 suggested an anthropogenic influence, as indicated by relatively high and positive loadings of atmospheric heavy metal emissions (i.e., HEAs, HECd, HECr, HEHg, HEPb), while PC2 was probably determined by Cd, Cu, DisInd*, and NDVI*. According to their relationships with the environmental factors, eight heavy metals could be clustered into five groups: (1) As, Cr, Ni, Zn, DisRd* and Pop; (2) Cd, Temp and NDVI*; (3) Cu, DisInd*, NDVI*, GDP, Pop, pH; (4) Pb and SOM; (5) Hg and DisRv*. This indicated that the population density and traffic activities mainly influenced the spatial variations in the As, Cr, Ni, and Zn pollution in soil, while the determination of sewage irrigation for Hg, temperature, and vegetation cover for Cd, the industrial activities and vegetation cover for Cu, and the soil properties for Pb. In addition, the soil physiochemical properties in terms of pH, clay, and sand* showed close correlations with the As, Cd, and Ni pollution in soil. At this scale, the effects of socioeconomic factors, human activities, and land covers seemed be apparent. The soil physicochemical properties were also pronounced as indicated by the relatively high correlation coefficients, but not prominently as anthropogenic factors (Table S3).

### 3.4. Cokriging Maps for Spatial Components

**Figure 4:**
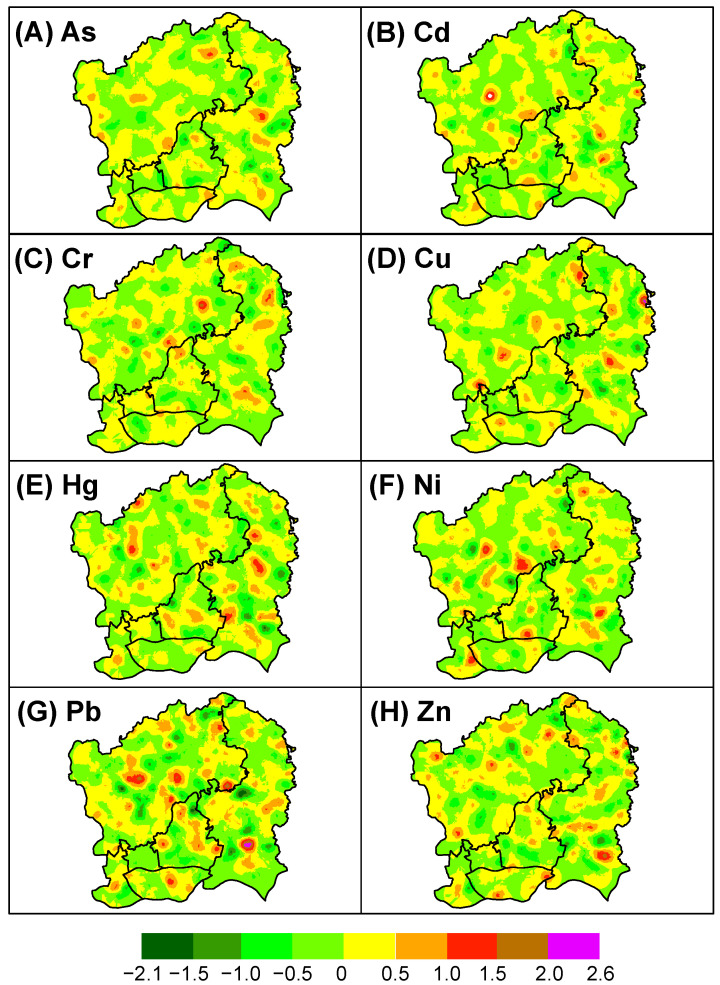
The cokriging maps of the spatial components for eight heavy metals at the short-range scale.

**Figure 5:**
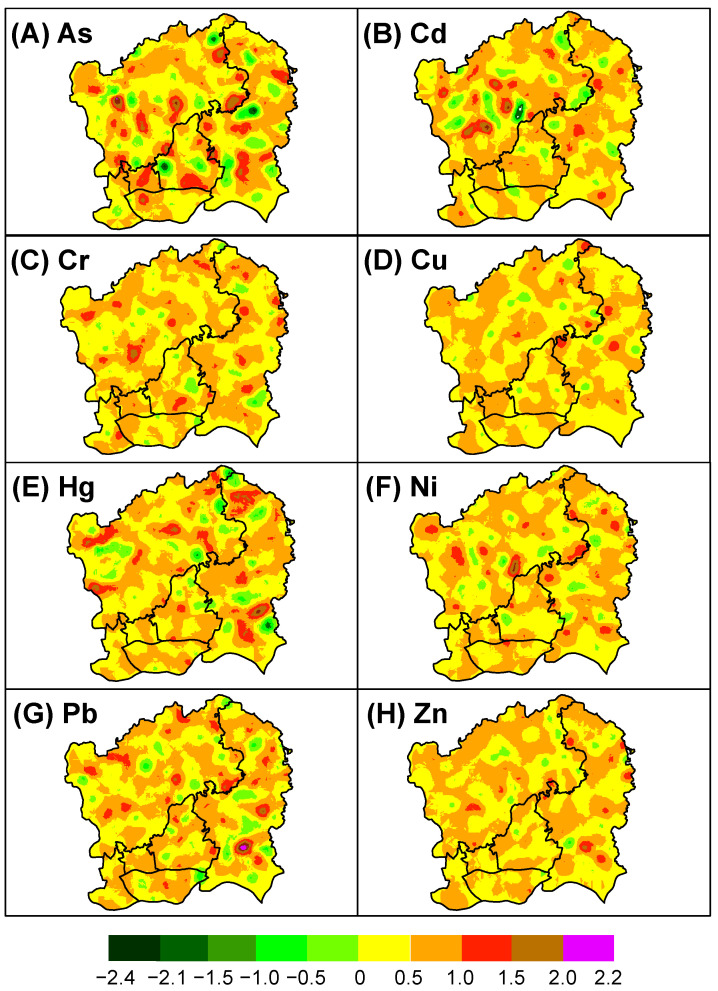
The cokriging maps of the spatial components for eight heavy metals at the long-range scale.

The spatial components of soil pollution for heavy metals in the same group by PCA on both the short and long structures showed quite similar patterns (Figure 4 and Figure 5). At the short structure, patches with high values of soil pollution (especially >0.4) were mainly scattered in northwestern (Baiyun District) and a few in central (Tianhe District), eastern (Huangpu District), and southern (Haizhu District) parts of the study area (Figure 4). High-value patches were highly localized and seemed to be clustered around potential point pollution sources such as industry and roads. At the long structure, the spatial component for soil pollution with heavy metals seemed more homogeneous across a large spatial range with small data ranges. Patches with large values at the long structure seemed to be more clustered, mainly in the northwestern and eastern urban areas (Figure 5).

## 4. Discussion

### 4.1. General Patterns of Soil Heavy Metal Pollution

Compared to the screening levels of heavy metal concentrations in agricultural soil (GB15618-2018) and development land (GB36600-218), less than 13% of the samples exceeded the threshold and might be hazardous to human health. The pollution exceeding rates were especially high for Cd, Pb, and As (4.7–12.6%), which is in line with previous studies [29,30]. Compared with previous studies in metropolitan areas in northern and southern China and other countries [31,32,33], the mean As, Cd, and Pb contents in the urban soil of Guangzhou were higher, while the mean values of Cr, Cu, Hg, and Ni were lower. In contrast, the Cu, Hg, Ni, and Zn contents of agricultural soils (i.e., soils in forest, orchard, and farmland) in the urban environment were higher in Guangzhou, while the mean contents of Cd, Cr and Pb were lower. A large variability of the soil pollution, and the bias of sampling sites and sizes could result in different results [1]. Cai et al. [34] reported a moderate to high pollution for most samples in urban soil as they collected samples from the southern Tianhe District (central part of our study area) and a certain land use type (i.e., urban land). Focusing on the whole of central Guangzhou and considering both agricultural and urban soils, we found that the average concentration levels of Cd, Cr, and Ni in our study were higher, but the Cu and Zn concentration levels were lower compared to Cai et al. [34]. To obtain the general spatial patterns of soil pollution, a large extent and random tessellation sampling design is needed given the high heterogeneity of soils in an urban environment.

### 4.2. Multi-Scale Variation of Soil Pollution

The FKA results clearly showed the spatial dependency of variance in soil heavy metal pollution. We found that patches with high pollution levels were mainly scattered in the Baiyun and Huangpu Districts of Guangzhou at the short-range scale, coinciding in areas of high densities of buildings, industries, and roads, similar to previous findings [29]. When filtering out the nugget effect and the short structure, changes in the spatial components for soil heavy metal pollution at the long structure tended to be more homogeneous across a large spatial range, as indicated by a small data range. Unlike ordinary kriging, which produces a single map, the FKA generated several cokriging maps for the spatial components of soil heavy metal pollution across scales. Although the spatial components did not have any physical meaning as a product of mathematics, they can help to understand the distinct soil pollution patterns and their underlying processes [14].

### 4.3. Scale-Dependent Drivers of Spatial Variations in Soil Pollution

The FKA results showed that the correlations between the soil heavy metal pollution and environmental factors were scale dependent. Mostly consistent with our hypothesis, the soil pollution was dominantly affected by the soil physiochemical properties on both the nugget effect and short-range scales, whereas socioeconomic factors and human activities such as traffic emissions dominated the variation in soil pollution on a long structure. The scale effect of environmental factors on soil heavy metal pollution, that is, a notable influence of the soil properties at local scales and pedogenesis/human activities at a large scale, was also found in other previous multi-scale studies [9,24,35]. As pointed out by [14], scale-dependent relationships are common in soil science and even the same physical process that controls the soil properties on different spatial scales might act in different ways.

At three scales, the soil physicochemical properties showed notable impacts on the soil heavy metal pollution, consistent with previous research [36,37,38]. The probable reason is that the soil properties reflected both the inputs of heavy metals at the local scale (e.g., pesticide and fertilizer addition) and the lithological legacies from the parent materials and the atmospheric deposition of anthropogenic activities at a large scale. Guangzhou is located in the central Pearl River Delta, an alluvial plain made up of alluvial deposits from three major upstream rivers. The upper reaches of the Xijiang and Beijiang Rivers are in the Nanling metallogenic belt [15]. The soil in the Pearl River Delta has inherited many heavy metals from alluvial deposits during pedogenisis [39,40]. Atmospheric deposition has been shown to be the main sources of heavy metals in both the urban and agricultural soils of China [41,42] and other regions [43,44,45]. Industry and transport emit large amounts of dust into the soil. The dust enriched with heavy metals accumulates in the soil through atmospheric deposition, causing changes in the soil properties such as pH, soil texture composition, and urban soil exchange capacity [46]. These changes favor the development of geochemical barriers where heavy metals affine [46]. For example, Cu and Zn favor the alkaline and chemisorption barriers, while As and Pb favor the chemisorption and organ mineral barriers. These geochemical barriers directly determine the mobility and storage capacity of soil heavy metals [46].

At the long structure, we found that the As, Cr, Ni, and Zn pollution in the soil was mainly influenced by human activities such as transport emissions and socioeconomic factors, while it was industrial emissions and NDVI for Cu, sewage irrigation for Hg, and the temperature and NDVI for Cd. Partly consistent with our findings, other researchers also found the traffic origins of Cr and Ni [7,47] and the anthropogenic origin of Hg in urban soil [37]. Close correlations of NDVI and Temp with soil heavy metal pollution have been shown in previous studies [16,48]. One probable reason is that the NDVI and Temp affected the heavy metal diffusion and migration by altering the soil physicochemical properties and subsequently changing the affinity of soil chemicals to heavy metal pollutants [48,49,50]. From this point of view, they seemed to exert more indirect influences. Cu, Pb, and Zn have often been traffic-related in urban environments in previous studies [51,52,53]. However, with a slight difference, we found that industrial emissions determined the soil pollution with Cu while it was the soil properties for Pb. As hypothesized, we thought there were positive correlations between the atmospheric emissions and soil pollution with Cr, Hg, and Pb at the long-range scale. However, we found that the correlations were relatively weak. One probable reason is that the atmospheric emissions in the study area were relatively homogeneous (Figure S2). Another reason could be that the atmospheric emissions do not equal the atmospheric deposition and their correlation was not linear. The process from aerial emissions to deposition was affected by many environmental factors such as precipitation, temperature, wind speed, and direction. Compared to the atmospheric emissions, distance factors such as DisRd* and DisInd* seemed to be more important in determining the distribution of soil heavy metals (e.g., Cr, Ni, and Cu). The complex correlations between the atmospheric emissions and soil heavy metal pollution need further study.

### 4.4. Implications for Soil Pollution Management at Multiple Scales

We found that the most notable influence of the soil physicochemical properties was on the soil heavy metal pollution at a short-range scale while it was less pronounced, but also an important influence at a long-range scale. This finding extends our current knowledge about the impacts of the soil properties on soil pollution, which was limited to a local scale. This is probably due to the characteristics of soil affecting the processes of heavy metals entering the soil at multiple scales, determining the accumulation rate and migration capacity of the metals in the soil. As the pH, SOM, and clay content increased, the soil tended to accumulate more heavy metals due to increased fixation, while it reduced the mobility of heavy metals to the soil [54,55]. Correspondingly, it will decrease the availability of heavy metals for plants and thus reduce their hazard. Thus, changing the soil properties could be an effective way to mitigation the pollution, of which the underlying mechanism is to reduce the mobility of heavy metals available for plants, and not their bulk concentrations in soil. In addition, we found highly negative correlations between soil Cd and Cu pollution and NDVI (positive with NDVI*) at a long-range scale. The distance to the pollutant sources had a notable influence on Cd and Hg pollution in the soil. This implies that planting trees, especially around the pollutant sources, would be effective in alleviating soil contaminants at long ranges because trees could capture or intercept the deposition of aerial pollutants [56,57]. However, tree species selection and the location of vegetation buffer designs in relation to pollution sources need further studies.

## 5. Conclusions

We explored the spatial variation of soil heavy metal pollution and its environmental drivers at multiple scales in a highly urbanized area of Guangzhou, South China using multivariate factorial kriging and based on 318 surface soil samples. Although the heavy metal contents for the majority of samples were elevated compared with the background values of Guangdong Province, less than 13% were hazardous to the ecosystem and humans. The unbiased probability-base sampling method suggested that soil heavy metal pollution in an urban environment of Guangzhou was not as high as reported. Factorial kriging analysis decomposed the spatial variation of soil pollution into a nugget effect, a short structure variance, and a long structure variance, with a decrease in the heterogeneity in the spatial variations as the spatial scale increased. Principal component analysis at each scale clearly discriminated the enhanced and counteractions of the correlations between the soil pollution and environmental factors at the three distinct scales. At both the nugget effect and short-range scales, soil pollution with nearly all the heavy metals was primarily influenced by soil physiochemical properties. At a long-range scale, the influences of anthropogenic factors were the most notable, while the soil physiochemical properties had less influence.

Our findings imply that the physicochemical properties of soil are good for predicting the soil heavy metal pollution at three scales, since they reflected both the local additions of heavy metals at a small scale, and the pedogenesis and anthropogenic inputs of heavy metals at a large scale. Multi-scale information derived from this current study could improve our understanding of the soil pollution mechanisms and thus be useful for pollution mitigation purposes. Improving the soil physicochemical properties of soil is effective at immobilizing the heavy metals in soil and thus reducing their availability for plants at both short and long distances. In addition, restoring vegetation including tree planting around pollutant sources as a nature-based solution (NBS) to solve pollution problems would be beneficial for alleviating soil pollution such as Cd, Cu, and Hg.

## Data Availability

Part of the environmental factor data provided in this manuscript can be accessed from the Institute of Geosciences and Resources, Chinese Academy of Sciences website (http://www.resdc.cn, accessed on 9 October 2018).

## Supplementary Materials

The following supporting information can be downloaded at: https://www.mdpi.com/article/10.3390/s22124496/s1, Figure S1: Variogram and cross-variogram maps for the eight heavy metals. Figure S2: Atmospheric emissions for heavy metals; Table S1: Statistics of the soil heavy metal pollution as indicated by the geo-accumulative indices (N = 318); Table S2: The general correlation coefficients among the soil contamination and environmental variables by the Pearson correlation analysis; Table S3: The structure correlation coefficients among the soil contamination and environmental variables.
