# Supplementary material for: Environmental Controls to Soil Heavy Metal Pollution Vary at Multiple Scales in a Highly Urbanizing Region in Southern China
Source: Sensors (Basel). 2022 Jun 14;22(12):4496. doi: 10.3390/s22124496 (PMC9229878; doi:10.3390/s22124496)
Supplement: Supplementary file 1 [file sensors-22-04496-s001.zip › sensors-1759169-supplementary.pdf]

Table S1. Statistics of soil heavy metal pollution as indicated by the geo-accumulative indices (N=318)

	Minimum	Maximum	Median	Mean	Standard deviation	Skewness	Kurtosis
$I_{geo}As$	-5.75	2.81	-0.70	-0.67	1.43	-0.18	-0.38
$I_{geo}Cd$	-5.49	6.49	1.91	1.90	1.48	0.07	3.33
$I_{geo}Cr$	-5.44	2.39	-0.97	-1.11	1.24	-0.77	1.07
$I_{geo}Cu$	-5.76	4.94	0.35	0.40	1.07	-0.14	4.84
$I_{geo}Hg$	-5.23	4.63	-0.45	-0.23	1.66	0.35	0.29
$I_{geo}Ni$	-4.82	4.37	-0.09	-0.25	1.05	-0.50	2.21
$I_{geo}Pb$	-2.59	3.32	0.10	0.05	0.78	0.15	1.51
$I_{geo}Zn$	-1.72	3.66	0.73	0.78	0.78	0.48	1.22

Table S2. General correlation coefficients among soil contamination and environmental variables by Pearson correlation analysis. Numbers in italic and bold represent significant at $p<0.05$.

	Igeo-							
	As	Cd	Cr	Cu	Hg	Ni	Pb	Zn
pH	***0.28***	***0.31***	0.01	***0.21***	-0.02	0.07	***0.30***	***0.29***
SOM	0.06	***0.29***	***0.25***	***0.27***	***0.41***	***0.43***	***-0.20***	***0.41***
Sand*	***0.41***	***0.19***	***0.49***	***0.26***	***0.35***	***0.59***	***0.30***	***0.34***
Silt	***0.16***	***0.31***	***0.196***	***0.29***	***0.15***	***0.23***	***0.16***	***0.40***
Clay	***0.16***	***0.19***	0.05	***0.26***	0.11	0.07	***0.19***	***0.24***
Prec	-0.07	-0.09	0.02	***-0.25***	0.02	***-0.13***	***-0.45***	***-0.30***
Temp	***0.35***	***0.33***	***0.32***	0.11	***0.36***	***0.17***	***-0.21***	0.09
GDP	***0.12***	***0.15***	0.03	***0.27***	0.06	***0.18***	***0.34***	***0.25***
Pop	***0.51***	***0.37***	***0.33***	***0.32***	***0.22***	***0.37***	***0.24***	***0.34***
DisRd*	***0.13***	***0.13***	0.04	0.06	0.03	0.09	-0.05	0.05
DsiRv*	0	0.1	0	-0.07	***0.17***	0.01	-0.04	-0.01
DisInd*	0.05	0.06	***0.13***	***0.17***	0.01	0.08	***0.15***	***0.13***
NDVI*	***0.41***	***0.41***	***0.29***	***0.35***	***0.22***	***0.26***	***0.16***	***0.33***
HEAs	***0.15***	0.09	***0.18***	***0.17***	0.05	***0.17***	***0.24***	***0.21***
HECd	***0.21***	***0.12***	***0.24***	***0.22***	0.09	***0.21***	***0.28***	***0.28***
HECr	***0.18***	0.1	***0.20***	***0.18***	0.08	***0.16***	***0.25***	***0.23***
HEHg	***0.34***	***0.24***	***0.26***	***0.27***	***0.16***	***0.27***	***0.24***	***0.31***
HEPb	***0.25***	***0.19***	***0.27***	***0.27***	***0.15***	***0.25***	***0.33***	***0.33***

Table S3. Structure correlation coefficients among soil contamination and environmental variables. The * represents the reciprocals of the variables.

	As	Cd	Cr	Cu	Hg	Ni	Pb	Zn
**Nugget effect**								
pH	-0.33	0.03	-0.25	-0.13	-0.26	-0.19	0.12	0.05
SOM	0.03	0.37	0.46	0.45	0.57	0.71	-0.07	0.65
Sand*	0.55	0.04	0.49	0.42	0.41	0.6	0.3	0.37
Silt	0.3	0.13	0.3	0.24	0.29	0.49	0.17	0.48
Clay	0.43	0.12	0.32	0.07	0.07	0.27	0.3	0.17
Prec	0.23	0.26	0.15	0.3	-0.04	0.13	0.51	0.35
Temp	-0.32	-0.53	-0.19	0.05	-0.25	-0.02	0	-0.17
GDP	0.28	0.05	0.34	-0.05	-0.01	0.15	-0.3	-0.02
Pop	-0.02	0.24	0.12	-0.06	0.06	0.15	0.01	0.15
DisRd*	0.04	0.28	-0.07	-0.04	-0.05	0.12	-0.05	0.16
DsiRv*	-0.15	-0.05	-0.05	-0.1	-0.21	0.05	-0.08	-0.14
DisInd*	-0.25	-0.02	-0.59	-0.49	-0.09	-0.25	-0.54	-0.12
NDVI*	0.1	0.2	0.32	0.15	0.1	0.19	0.45	0.21
HEAs	0.11	0.07	-0.05	0.17	-0.11	-0.13	0.33	0.05
HECd	0.04	-0.17	-0.26	-0.19	0.01	-0.33	-0.02	-0.26
HECr	0.31	0.09	0.19	0.18	-0.08	0.04	0.54	0
HEHg	-0.42	-0.5	-0.43	-0.31	-0.13	-0.38	-0.23	-0.31
HEPb	-0.02	-0.22	-0.22	-0.35	-0.16	-0.32	-0.4	-0.36
**Short structure (3km)**								
pH	0.57	0.5	0.38	0.56	0.06	0.27	0.49	0.6
SOM	0.44	0.33	-0.12	-0.03	0.1	0.12	-0.41	0.31
Sand*	0.19	0.27	0.33	0.06	0.03	0.39	0.53	0.09
Silt	0.03	0.3	0.11	0.06	-0.27	0.06	-0.03	0.3
Clay	0.4	0.12	-0.13	0.5	0.25	-0.16	-0.04	0.4
Prec	-0.37	-0.27	-0.33	-0.32	-0.16	-0.3	-0.05	-0.37
Temp	0.04	0.11	0.06	-0.31	0.02	-0.04	-0.14	-0.09
GDP	-0.61	-0.56	-0.11	-0.43	-0.27	-0.15	-0.55	-0.52
Pop	-0.59	-0.67	-0.11	-0.23	-0.44	-0.38	-0.21	-0.3
DisRd*	-0.34	-0.54	-0.28	-0.02	0.11	-0.58	0.03	-0.41
DsiRv*	0.21	0.11	0.08	0.11	0.4	-0.16	0.24	0.22
DisInd*	-0.05	-0.22	0.02	-0.04	0.03	-0.03	0.25	-0.05
NDVI*	-0.63	-0.48	-0.51	-0.38	-0.7	-0.3	-0.4	-0.4
HEAs	-0.14	-0.15	-0.35	-0.47	0.05	-0.1	-0.57	-0.49
HECd	-0.05	-0.01	-0.27	-0.35	0.05	-0.14	-0.31	-0.26
HECr	0.05	-0.07	-0.46	-0.21	0.13	-0.18	-0.46	-0.23
HEHg	-0.15	0.02	0	-0.3	-0.02	-0.02	0	-0.04
HEPb	-0.06	0	-0.15	-0.35	0.14	-0.1	-0.2	-0.22
**Long structure (12km)**								
pH	0.32	0.46	0.29	0.45	0.28	0.1	0.18	0.19

SOM	-0.41	-0.01	-0.46	-0.19	0.25	-0.28	-0.35	-0.41
Sand*	0.25	0.07	0.24	-0.2	0.26	0.59	0.17	0.3
Silt	-0.05	0.23	-0.11	0.4	0.15	-0.31	0.07	0.12
Clay	-0.1	0.21	-0.28	0.4	0.13	-0.4	0.1	0.16
Prec	0.05	-0.06	0.08	-0.27	0.13	-0.07	-0.41	-0.37
Temp	0.28	0.62	0.35	0.43	0.57	0.23	-0.18	0.25
GDP	0.04	0.32	0.04	0.46	0.2	0.18	0.43	0.42
Pop	0.44	0.48	0.44	0.47	0.26	0.57	0.2	0.35
DisRd*	0.41	0.16	0.49	0.4	-0.17	0.49	-0.13	0.08
DsiRv*	-0.05	0.35	-0.16	-0.19	0.71	0.16	-0.1	0.24
DisInd*	0.05	0.26	0.56	0.68	-0.02	0.2	-0.05	0.2
NDVI*	0.45	0.73	0.38	0.7	0.47	0.2	0.08	0.48
HEAs	-0.03	-0.07	0.25	0.1	-0.07	0.14	0.16	0.18
HECd	0.04	-0.08	0.31	0.26	-0.12	0.23	0.18	0.3
HECr	-0.02	-0.11	0.29	0.12	-0.11	0.16	0.26	0.24
HEHg	0.13	0.16	0.21	0.31	0.04	0.2	-0.1	0.18
HEPb	0.05	0.07	0.35	0.35	0.03	0.28	0.24	0.38

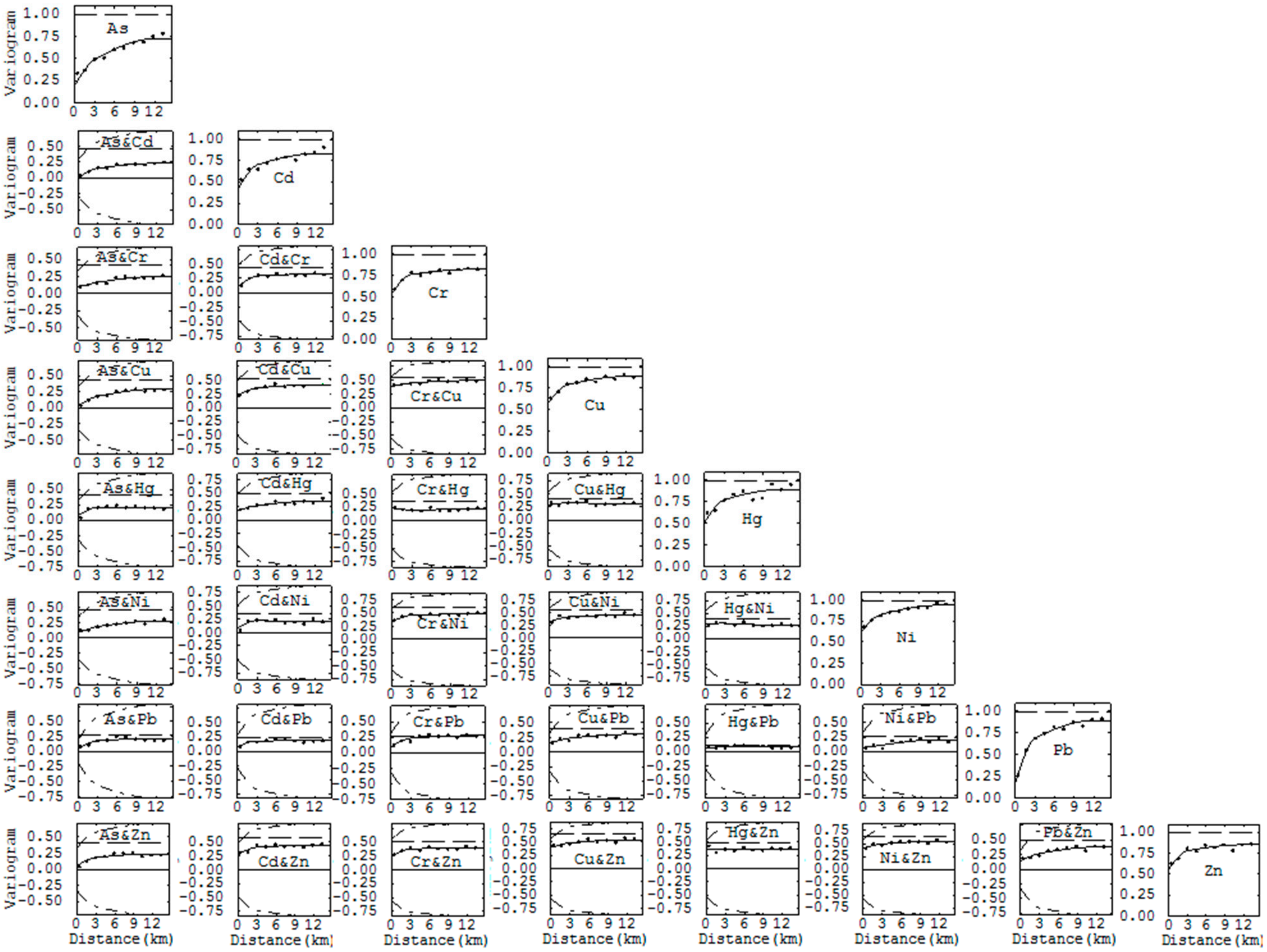

Figure S1. Variogram and cross-variogram maps for the eight heavy metals. The plotted points, solid lines, dash-dotted lines, and dashed lines represent the experimental values, the model of coregionalization and the hull of perfect correlation, and the experimental variances, respectively.

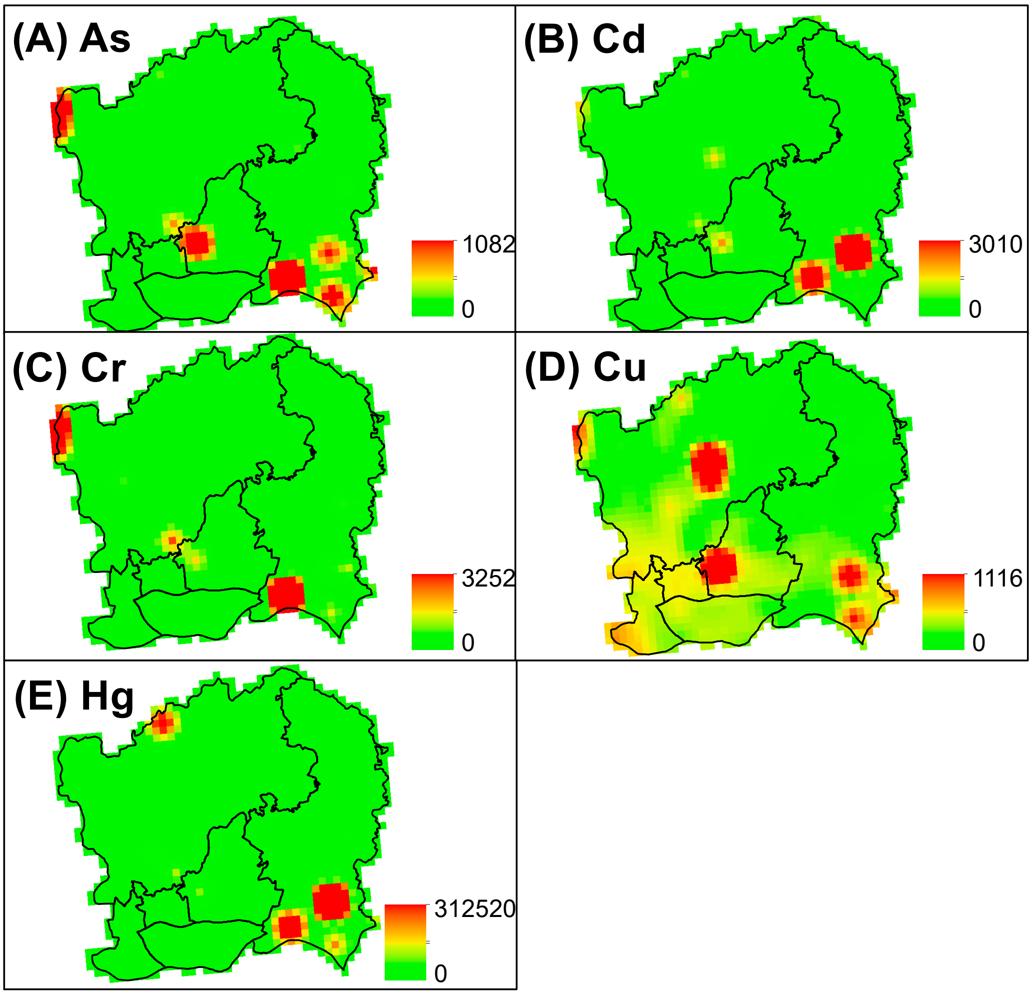

Figure S2. Atmospheric emissions for heavy metals (Unit: g)
